# Long-term trends of tuberculosis incidence and mortality in four central African countries

**DOI:** 10.1038/s41598-021-95967-8

**Published:** 2021-08-17

**Authors:** Nodjimadji Tamlengar Martial, Sumaira Mubarik, Chuanhua Yu

**Affiliations:** Department of Epidemiology and Biostatistics, School of Health Sciences, Wuhan University, Wuhan, 430071 China

**Keywords:** Infectious diseases, Epidemiology

## Abstract

Tuberculosis (TB) incidence and mortality rates are still high in Sub-Saharan Africa, and the knowledge about the current patterns is valuable for policymaking to decrease the TB burden. Based on the Global Burden of Disease (GBD) study 2019, we used a Joinpoint regression analysis to examine the variations in the trends of TB incidence and mortality, and the age-period-cohort statistical model to evaluate their risks associated with age, period, and cohort in males and females from Cameroon (CAM), Central African Republic (CAR), Chad, and the Democratic Republic of the Congo (DRC). In the four countries, TB incidence and mortality rates displayed decreasing trends in men and women; except for the males from DRC that recorded an almost steady pattern in the trend of TB incidence between 1990 and 2019. TB incidence and mortality rates decreased according to the overall annual percentage changes over the adjusted age category in men and women of the four countries, and CAM registered the highest decrease. Although TB incidence and mortality rates increased with age between 1990 and 2019, the male gender was mainly associated with the upward behaviors of TB incidence rates, and the female gender association was with the upward behaviors of TB mortality rates. Males and females aged between 15–54 and 15–49 years old were evaluated as the population at high risks of TB incidence and mortality respectively in CAM, CAR, Chad, and DRC. The period and cohort relative risks (RRs) both declined in men and women of the four countries although there were some upward behaviors in their trends. Relatively to the period and cohort RRs, females and males from CAM recorded the most significant decrease compared to the rest of the countries. New public health approaches and policies towards young adults and adults, and a particular focus on elderlies’ health and life conditions should be adopted in CAM, CAR, DRC, and Chad to rapidly decrease TB incidence and mortality in both genders of the four countries.

## Introduction

Labeled as a global disease, tuberculosis (TB) is one of the most dangerous infectious diseases in the world, causing the death of millions of people every year^1–3^. TB is caused by the bacillus *Mycobacterium tuberculosis*, the single infectious agent^1–4^. TB spreads generally through the air from one person to another, giving it the qualification of an airborne disease, and it typically affects lungs where it lodges after inhalation and can affect other organs following hematogenous distribution such as the brain, kidneys, lungs, spine, and intestines^4^.

The African continent had made a progress by decreasing TB incidence and mortality by nearly 16 and 19 percent (16% and 19%) respectively during 2020^5^. The Democratic Republic of the Congo (DRC), the Central African Republic (CAR), Cameroon (CAM), and Chad are four countries, located in the central region of Africa^6^. During 2017, almost 50% of the population in each of these countries was female^7^. The Human Development Index (HDI) is estimated by a three-dimensional index evaluation such as the life expectancy index, education index, and economic growth index. It reflects the growth of the country and human development in terms of a healthy lifestyle and reasonable standards of living in general^8^. Between 1990 and 2019, United Nations Development Programme (UNDP) listed countries according to their Human Development Index (HDI), and only CAM had a continuous growth from 1990; meanwhile Chad had a declining pattern of the HDI since 2015^9^. DRC and CAR registered increasing HDI values during 2000 and 2010 accordingly^9^.

In the central region of Africa, TB incidence rates were between 100 and 199 per 100,000 people in CAM and Chad, and between 300 and 499 per 100,000 people in DRC during 2019^5^. In CAR instead, the TB incidence rate was higher than 500 per 100,000 people across the same year^5^. Within the same year (2019), TB mortality rates per 100,000 people were estimated at around 22 in Chad, 29 in CAM, 49 in DRC, and 98 in CAR^10^.

Many studies on TB addressed various aspects of TB incidence and mortality in Chad, CAM, DRC, and CAR^11–17^. However, studies outlining the status of the updated trends of TB incidence and mortality, and risks associated with age, period, and birth cohort in the four countries from 1990 to 2019 by gender have not yet been presented. Our research objective is therefore to investigate the patterns in the trends of TB incidence and mortality from 1990 to 2019, and their association with age, period, and birth cohort as relative risks in the male and female populations of CAM, CAR, Chad, and DRC. Initiated because of the sustainable development goals (SDGs) set by the United Nations (UN), the “End TB pandemic strategy” was advocated in 2014 for the period 2016–2035 by the world health assembly. The “End TB pandemic strategy”, on one hand, is based on the decrease of TB mortality rates by 90% in 2030 compared to 2015 during 2030, followed by a reduction of 95% for the year 2035. On the other hand, TB incidence was estimated to be reduced by 80% and 90% for the years 2030 and 2035 respectively^18^. Examining the long-term trends of TB incidence and mortality, and investigating their risks related to age, period, and birth cohort might help to decrease the burden of TB disease and reach the “End TB pandemic strategy” set by the World Health Organization (WHO).

## Materials and methods

### Data source

All TB incidence and mortality statistics and the population data used in the study were extracted from the Global Burden of Diseases (GBD) 2019^19^. They represent all causes of TB disease. The GBD database is available publicly and controlled by the Institute for Health Metrics and Evaluation (IHME) and is based in Seattle, Washington State, USA. Generally, household surveys with complete summary birth histories, censuses, vital registration, disease surveillance system, and sample registration systems constitute the primary data input for the GBD. The data used for our analysis are in GBD data input in the Global Health Data exchange (GHDx) section in the result tools. Rates were estimated as per 100,000 people. Ethical approval was not needed for this study because there was no direct involvement of human subjects.

### Statistical analysis

TB incidence and mortality outcomes were evaluated through an age-period-cohort statistical model analysis. The age effects represent different risks during different periods of life. Period effects indicate the population-large exposure at a specific point of time, and different risks in different birth cohorts are reflected by cohort effects mainly^20,21^. To isolate the different contributions of age, period, and cohort, we decomposed the age and cycled a queue into their linear and nonlinear constituents^22^. This decomposition also produced many essential functions such as net drift, local drifts, longitudinal age trend, period, and cohort deviations^23^. The local drifts represent the annual percentage changes in each age group, and the net drift represents the overall annual percentage changes of the adjusted age group over time. The longitudinal age curve is adjusted for period deviations and represents the fitted longitudinal age-specific rates in the reference cohort. The period relative risk (RR) defines the period RR adjusted for age and nonlinear cohort contributions of each period relative to the reference period. The cohort relative risk (RR) defines the cohort RR adjusted for age and nonlinear period contributions in each cohort relative to the reference period^24^. Incidence rates, mortality rates, and demographic statistics were decomposed into five consecutive years before conducting our Age-Period-Cohort (APC) analysis. They were arranged from 1990–1994 (median 1992.5) to 2015–2019 (median 2017.5). Consecutive five years age groups were set from 15–19 (median 17.5) to 75–79 (median 77.5). The birth cohort arrangements concerned those born from 1975 to 2004 and were also divided into 5 consecutive years for the APC analysis. The reference values were selected as the lower two central values in the event of an even number of categories. Wald chi-square tests were conducted with a *p*-value set at *p* < 0.01 for statistical significance. Despite the APC analysis advantages, APC possesses limitations such as uncertainty principle and identifiability problems^24^. The uncertainty principle indicates the measurements of absolute rates in cohorts that are not frequently taken into consideration by most epidemiological cohort and case–control researchers^24^. The identifiability problem is the fact that the three scales of age, period and cohort are collinear from the equation cohort equals period minus age; thus, the log-linear trends in the rates cannot only be representing the contributions of age, period, and cohort^21^. We used the APC Web Tool (Biostatistics Branch, National Cancer Institute, Bethesda, MD, USA) software in our statistical analysis.

Through a Joinpoint regression analysis, we investigate the variations in the trends of TB incidence and mortality in, CAM, CAR, Chad, and DRC by gender during 1990–2019. Years with significant changes in the patterns were identified, and the annual percentage change (APC) and the average annual percent change (AAPC) along with their 95% confidence interval (CI) were also estimated for each trend segment from 1990 to 2019. We utilized the Joinpoint regression program version 4.8.0.1 (April 2020) from the Statistical Research and Application branch of the Surveillance Research Program of the United States (U.S) Nation Cancer Institute to run the Joinpoint regression analysis.

## Results

### The trends of tuberculosis crude and age-standardized incidence and mortality rates in Cameroon, Chad, Central African Republic, and the Democratic Republic of the Congo by gender

**Figure 1:**
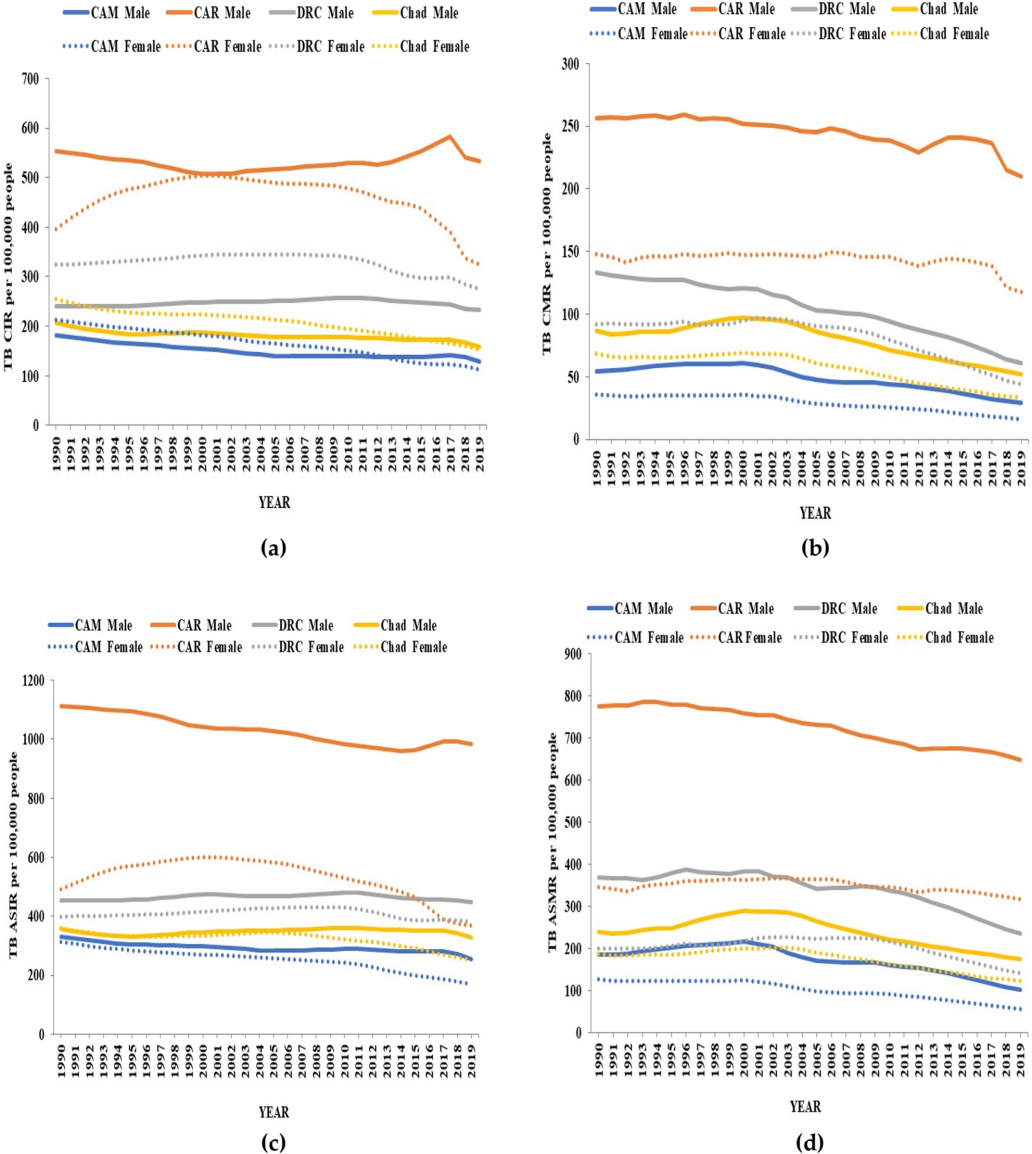
The Trends of Tuberculosis crude incidence rate (TB CIR) (**a**), Tuberculosis crude mortality rate (TB CMR) (**b**), Tuberculosis age-standardized incidence rate (TB ASIR) (**c**), and Tuberculosis age-standardized mortality rate (TB ASMR) (**d**) by gender in Cameroon (CAM), Central African Republic (CAR), the Democratic Republic of the Congo (DRC), and Chad.

Figure 1 represented the temporal progression of TB crude and age-standardized incidence and mortality rates in the four countries by gender from 1990 to 2019. All trends displayed decreasing or steady patterns in males and females of the four countries. The trends of TB crude incidence rate (CIR) and the age-standardized incidence rate (ASIR) displayed similar patterns (a and b), as well as the trends of TB crude mortality rate (CMR) and the age-standardized mortality rate (ASMR) from 1990 to 2019 (c and d). TB ASMR was lower than the TB ASIR in both sexes, and the patterns representing the male gender are above those representing the female gender in all four countries. CAR recorded the highest and CAM the lowest TB ASIR and ASMR in males and females compared to the rest of the countries.

### The estimated trends of TB incidence and mortality by gender in Cameroon, Chad, Central African Republic, and the Democratic Republic of the Congo using Joinpoint regression analysis

Tables 1 and 2 described the annual percentage change (APC) and the average annual percent change (AAPC) of the age-standardized TB incidence (Table 1) and mortality (Table 2) in males and females of CAM, CAR, DRC, and Chad between 1990 and 2019, from our Joinpoint regression analysis. The APC of TB incidence and mortality in males and females showed substantial changes in the four countries during some periods in our study.

**Table 1:** Trends of Tuberculosis age-standardized incidence rate by gender in Cameroon (CAM), Central African Republic (CAR), the Democratic Republic of the Congo (DRC), and Chad from 1990 to 2019.

Segments	CAM		CAR		DRC		Chad	
	Year	APC (95% CI)	Year	APC (95% CI)	Year	APC (95% CI)	Year	APC (95% CI)
**ASIR male**								
Trend 1	1990–1993	− 2.1* (− 2.9, − 1.3)	1990–1996	− 0.4* (− 0.4, − 0.3)	1990–1995	0.1 (− 0.0, 0.2)	1990–1992	− 2.3* (− 2.6, − 2.0)
Trend 2	1993–2006	− 0.7* (− 0.8, − 0.6)	1996–2000	− 1.1* (− 1.4, − 0.9)	1995–2000	0.8* (0.6, 1.0)	1992–1995	− 1.0* (− 1.3, − 0.7)
Trend 3	2006–2011	0.4 (− 0.1, 0.9)	2000–2005	− 0.2* (− − 0.4, − 0.1)	2000–2005	− 0.3* (− 0.5, − 0.1)	1995–2000	1.0* (0.9, 1.1)
Trend 4	2011–2014	− 1.1 (− 2.6, 0.5)	2005–2014	− 0.8* (− 0.9, − 0.8)	2005–2010	0.7* (0.5, 0.9)	2000–2010	0.4* (0.3, 0.4)
Trend 5	2014–2017	0.3 (− − 1.3, 1.9)	2014–2017	1.2 (− 0.8, 1.7)	2010–2014	− 1.0* (− 1.3, − 0.7)	2010–2017	− 0.4* (− 0.4, − 0.3)
Trend 6	2017–2019	− 5.0* (− 6.5, − 3.5)	2017–2019	− 0.3 (− 0.7, 0.2)	2014–2019	− 0.7* (− 0.8, − 0.5)	2017–2019	− 3.0* (− 3.3, − 2.7)
AAPC	1990–2019	− 0.9* (− 1.2, − 0.6)	1990–2019	− 0.4* (− 0.5, − 0.4)	1990–2019	− 0.0 (− 0.1, 0.0)	1990–2019	− 0.3* (− 0.3, − 0.2)
**ASIR female**								
Trend 1	1990–1994	− 2.2* (− 2.3, − 2.1)	1990–1994	3.5* (3.3, 3.7)	1990–1996	0.3* (0.1, 0.5)	1990–1994	− 2.0* (− 2.3, − 1.6)
Trend 2	1994–2008	− 1.0* (− 1.0, − 1.0)	1994–2000	1.0* (0.9, 1.1)	1996–2004	0.7* (0.5, 0.8)	1994–1997	0.0 (− 0.3, 0.3)
Trend 3	2008–2011	− 1.8* (− 2.1, − 1.5)	2000–2005	− 0.5* (− 0.7, − 0.4)	2004–2008	0.3 (− 0.2, 0.8)	1999–2005	0.7* (0.4, 0.9)
Trend 4	2011–2014	− 4.5* (− 4.7, − 4.2)	2005–2014	− 2.0* (− 2.0, − 1.9)	2008–2011	− 0.4 (− 1.5, 0.7)	2005–2008	− 1.2* (− 2.1, − 0.2)
Trend 5	2014–2017	− 3.4* (− 3.6, − 3.1)	2014–2017	− 7.1* (− 7.7, − 6.6)	2011–2014	− 2.8* (− 3.9, − 1.8)	2008–2014	− 1.7* (− 2.0, − 1.5)
Trend 6	2017–2019	− 4.8* (− 5.0, − 4.5)	2017–2019	− 3.5* (− 4.0, − 2.9)	2014–2019	− 0.5* (− 0.7, − 0.3)	2014–2019	− 3.5* (− 3.8, − 3.3)
AAPC	1990–2019	− 2.1* (− 2.2, − 2.1)	1990–2019	− 1.0* (− 1.1, − 1.0)	1990–2019	− 0.1 (− 0.3, 0.0)	1990–2019	− 1.2* (− 1.4, − 1.1)

*ASIR* age-standardized incidence rate, *APC* annual percentage change, *AAPC* average annual percent change, *CI* confidence interval.

*Values significantly different from zero at alpha = 0.05.

**Table 2:** Trends of Tuberculosis age-standardized mortality rate by gender in Cameroon (CAM), Central African Republic (CAR), the Democratic Republic of the Congo (DRC), and Chad from 1990 to 2019.

Segments	CAM		CAR		DRC		Chad	
	Year	APC (95% CI)	Year	APC (95% CI)	Year	APC (95% CI)	Year	APC (95% CI)
**ASMR male**								
Trend 1	1990–1998	1.9* (1.6, 2.1)	1990–1994	0.3* (0.1, 0.6)	1990–1993	− 0.5 (− 1.7, 0.7)	1990–1992	− 0.5 (− 1.3, 0.3)
Trend 2	1998–2001	0.2 (− 1.7, 2.1)	1994–1999	− 0.5* (− 0.7, − 0.2)	1993–1996	1.7 (− 0.7, 4.2)	1992–1995	2.0* (1.1, 2.8)
Trend 3	2001–2005	− 5.6* (− 6.5, − 4.7)	1999–2006	− 0.8* (− 0.9, − 0.6)	1996–2001	− 0.1 (− 0.8, 0.7)	1995–2000	3.0* (2.7, 3.3)
Trend 4	2005–2009	0.5 (− 1.4, 0.5)	2006–2012	− 1.2* (− 1.4, − 1.0)	2001–2005	− 2.6* (− 3.7, − 1.4)	2000–2003	− 0.7 (− 1.5, 0.1)
Trend 5	2009–2014	− 3.0* (− 3.6, − 2.4)	2012–2016	− 0.1 (− 0.5, 0.4)	2005–2010	0.1 (− 0.7, 0.9)	2003–2010	− 3.5* (− 3.7, − 3.4)
Trend 6	2014–2019	− 6.7* (− 7.1, − 6.3)	2016–2019	− 1.2* (− 1.7, − 0.8)	2010–2019	− 4.0* (− 4.3, − 3.8)	2010–2019	− 2.5* (− 2.6, − 2.5)
AAPC	1990–2019	− 2.0* (− 2.3, − 1.8)	1990–2019	− 0.6* (− 0.7, − 0.5)	1990–2019	− 1.5* (− 1.8, − 1.2)	1990–2019	− 1.1* (− 1.2, − 0.9)
**ASMR female**								
Trend 1	1990–1992	− 1.6* (− 3.3, − 0.0)	1990–1992	− 1.0 (− 2.4, 0.3)	1990–1999	0.7* (0.4, 0.9)	1990–1992	− 1.1* (− 1.6, − 0.6)
Trend 2	1992–2001	0.1 (− 0.1, 0.3)	1992–1996	1.7* (1.0, 2.4)	1999–2002	2.7 (− 0.2, 5.8)	1992–1996	0.6* (0.4, 0.9)
Trend 3	2001–2005	− 5.6* (− 6.3, − 4.8)	1996–2005	0.1 (− 0.0, 0.3)	2002–2005	− 0.9 (− 3.7, 2.1)	1996–1999	2.1* (1.6, 2.6)
Trend 4	2005–2010	− 1.4* (− 1.9, − 0.8)	2005–2012	− 1.2* (− 1.4, − 1.0)	2005–2008	0.6 (− 2.3, 3.6)	1999–2003	0.4* (0.1, 0.6)
Trend 5	2010–2014	− 3.9* (− 4.7, − 3.1)	2012–2015	0.2 (− 1.4, 1.6)	2008–2011	− 2.7 (− 5.5, 0.2)	2003–2008	− 2.9* (− 3.0, − 2.7)
Trend 6	2014–2019	− 6.4* (− 6.8, − 6.1)	2015–2019	− 1.6* (− 2.0, − 1.1)	2011–2019	− 4.7* (− 5.0, − 4.4)	2008–2019	− 3.2* (− 3.2, − 3.2)
AAPC	1990–2019	− 2.8* (− 3.0, − 2.6)	1990–2019	− 0.3* (− 0.5, − 0.1)	1990–2019	− 1.2* (− 1.7, − 0.6)	1990–2019	− 1.4* (− 1.5, − 1.4)

*ASMR* age-standardized mortality rate, *APC* annual percentage change, *AAPC* average annual percent change, *CI* confidence interval.

*Values significantly different from zero at alpha = 0.05.

TB incidence decreased significantly in men and women of the four countries according to their respective AAPC, except for males in DRC that recorded an almost steady behavior in the trend of TB incidence from 1990 to 2019 based on the findings of the Joinpoint regression analysis (Table 1). TB incidence decreased the most in males and females of CAM (0.9% and 2.1% respectively) compared to the rest of the countries. The declining trends of TB incidence in men and women of DRC were the lowest amongst the four countries (− 0.0% and − 0.1% respectively) from 1990 to 2019.

In men and women from CAM, CAR, DRC, and Chad, TB mortality had declined significantly during 1990–2019. The decrease of TB mortality in males and females from CAM was the highest compared to CAR, DRC, and Chad. The decrease was nearly 2.0% and 2.8% in men and women from CAM respectively. In CAR instead, TB mortality decrease was the lowest in males as well as in females with nearly 0.6% and 0.3% respectively compared to the other countries as displayed in Table 2.

### The net drifts and local drifts of TB incidence mortality by gender in Cameroon, Chad, Central African Republic, and the Democratic Republic of the Congo

**Figure 2:**
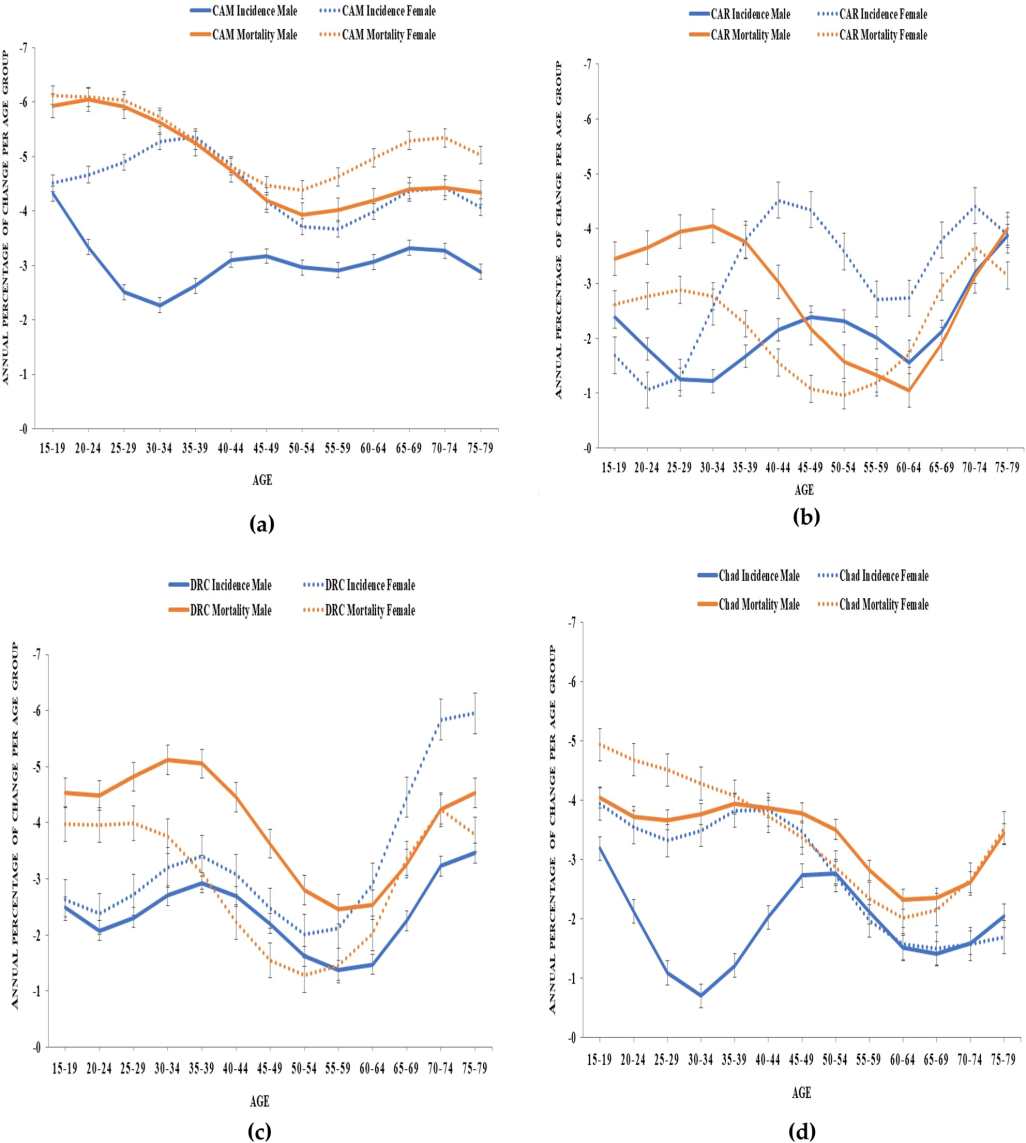
The Trends of the local drifts of TB incidence mortality in each age group in males and females from Cameroon (CAM) (**a**), Central African Republic (CAR) (**b**), the Democratic Republic of the Congo (DRC) (**c**), and Chad (**d**) with their 95% confidence intervals.

The local drifts of TB incidence and mortality by gender in each of the four countries are represented in Fig. 2. The local drifts are the annual percentage of changes in each age group from 15–19 to 75–79 years old. All local drift values were below zero in both TB incidence and mortality across the four countries in males and females, indicating a decline.

Despite the decreasing pattern of TB incidence in different age groups of men and women from the four countries, the trends of the local drifts registered upward behaviors in some age groups. Almost all age groups in men and women were associated with the upward behaviors in the patterns of the local drifts of all the countries, except for the females from Chad that recorded the upward behavior only in women older than 50 years old.

Based on the local drifts of TB mortality in the populations of the four countries, upward behaviors in the patterns of the annual percentage changes of each age groups could be observed in adults’ males and females older than 35 years from the four countries, except for Chad where it had been associated with young males older than 25 years old. The upward behaviors in the trends of the local drifts of TB incidence mortality were associated with both genders in all four countries during different age groups.

Table 3 displayed the net drifts of the TB incidence and mortality in men and women of CAM, CAR, DRC, and Chad. The net drift is defined as the overall annual percentage changes over time for the adjusted age group (15–79 years old). Females and males from CAM recorded the highest value of the net drifts (highest decrease); meanwhile, men and women from Chad had the lowest decrease (lowest net drift values) of TB incidence compared to the other countries.

**Table 3:** Net drifts of tuberculosis incidence and mortality rates by gender in Cameroon (CAM), Central African Republic (CAR), the Democratic Republic of the Congo (DRC), and Chad.

Variables	Gender	CAM	CAR	DRC	Chad
		Net drift (95% CI)	Net drift (95% CI)	Net drift (95% CI)	Net drift (95% CI)
TB ASIR	Male	− 2.99 (− 3.21; − 2.76)	− 2.07 (− 2.3; − 1.75)	− 2.29 (− 2.42; − 2.16)	− 1.89 (− 2.05; − 1.73)
	Female	− 4.45 (− 4.63; − 4.26)	− 3.26 (− 3.50; − 3.01)	− 3.12 (− 3.26; − 2.99)	− 2.88 (− 3.04; − 2.88)
TB ASMR	Male	− 4.74 (− 5.07; − 4.42)	− 2.70 (− 3.11; − 2.29)	− 3.90 (− 4.14; − 3.66)	− 3.40 (− 3.66; − 3.14)
	Female	− 5.12 (− 5.58; − 4.65	− 2.06 (− 2.26; − 1.85)	− 2.71 (− 3.00; − 2.41)	− 3.39 (− 3.76; − 3.03)

*TB* tuberculosis, *ASIR* age-standardized incidence rate, *ASMR* age-standardized mortality rate, *CI* confidence interval.

Oppositely, in men and women aged from 15 to 79 years old, CAM registered the highest net drift values of TB mortality, and CAR had the lowest values compared to the rest of the countries.

Comparing the two genders, TB incidence decreased mostly in the female gender compared to the male gender in all four countries according to the values of the net drifts. Concerning TB mortality, the females from CAM and Chad had a higher decrease compared to males. Males from CAR and DRC are associated with the decrease of TB mortality compared to the females according to their respective net drift values.

### The age relative risks of TB incidence and mortality by gender in Cameroon, Chad, Central African Republic, and the Democratic Republic of the Congo

**Figure 3:**
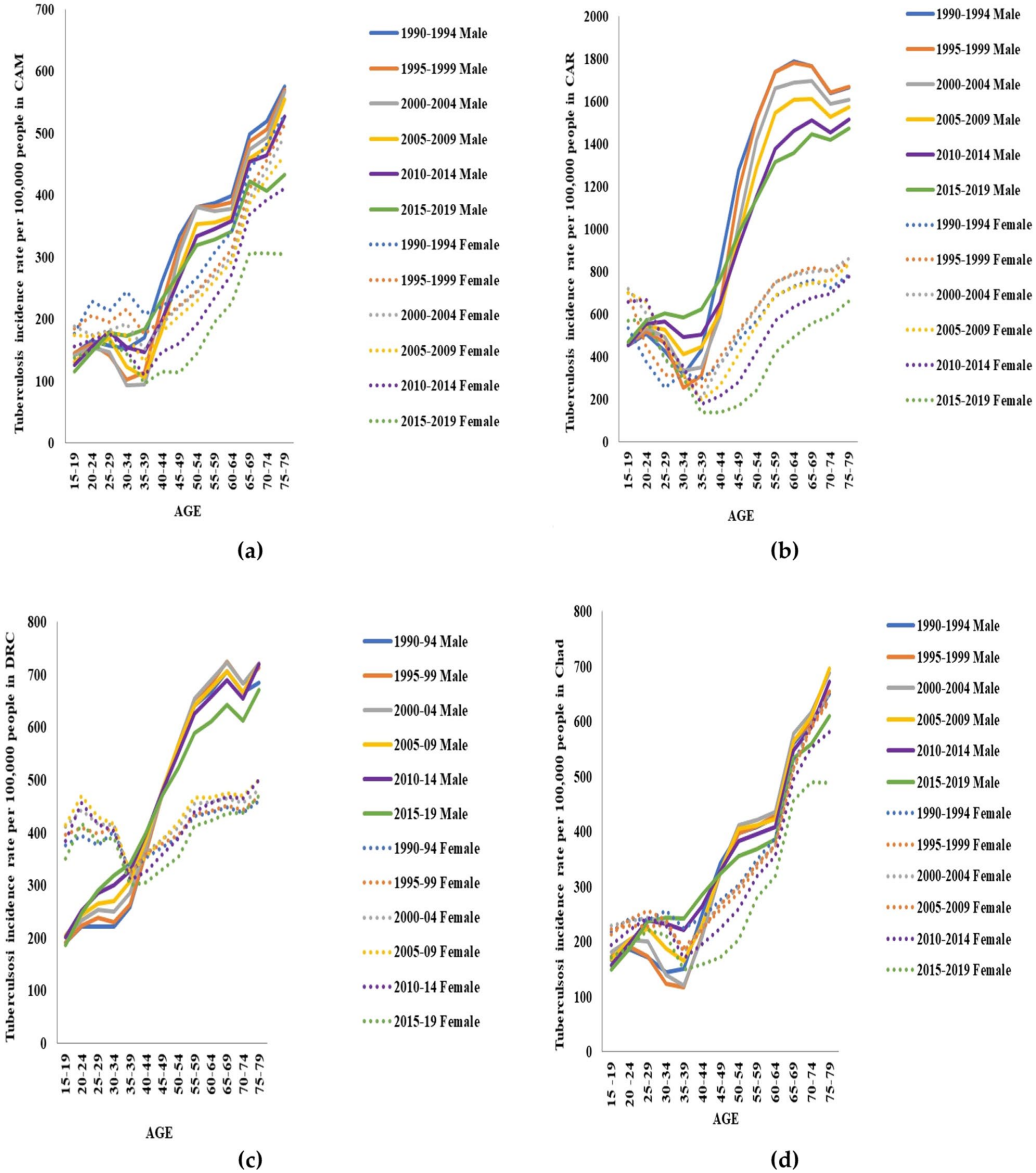
Tuberculosis incidence rates in each age group in males and females from Cameroon (CAM) (**a**), Central African Republic (CAR) (**b**), the Democratic Republic of the Congo (DRC) (**c**), and Chad (**d**) during 1990–2019.

According to age, TB incidence in males and females from CAM, CAR, DRC, and Chad displayed increasing trends with up and downward behaviors. TB incidence rates increased and reached their peak point in individuals aged between 75 and 79 years in all men and women from the four countries. TB incidence rates per age group during 1990–2019 showed that in CAM and Chad, the trends displayed similar patterns in males and females. The same finding could be noticed in the trends representing each age group in men and women from CAR and DRC. All TB incidence rates during 2015–2019 in all age groups were the lowest compared to other years. Furthermore, TB incidence rates in each age group in the male gender were higher than those in the female gender as represented in Fig. 3.

**Figure 4:**
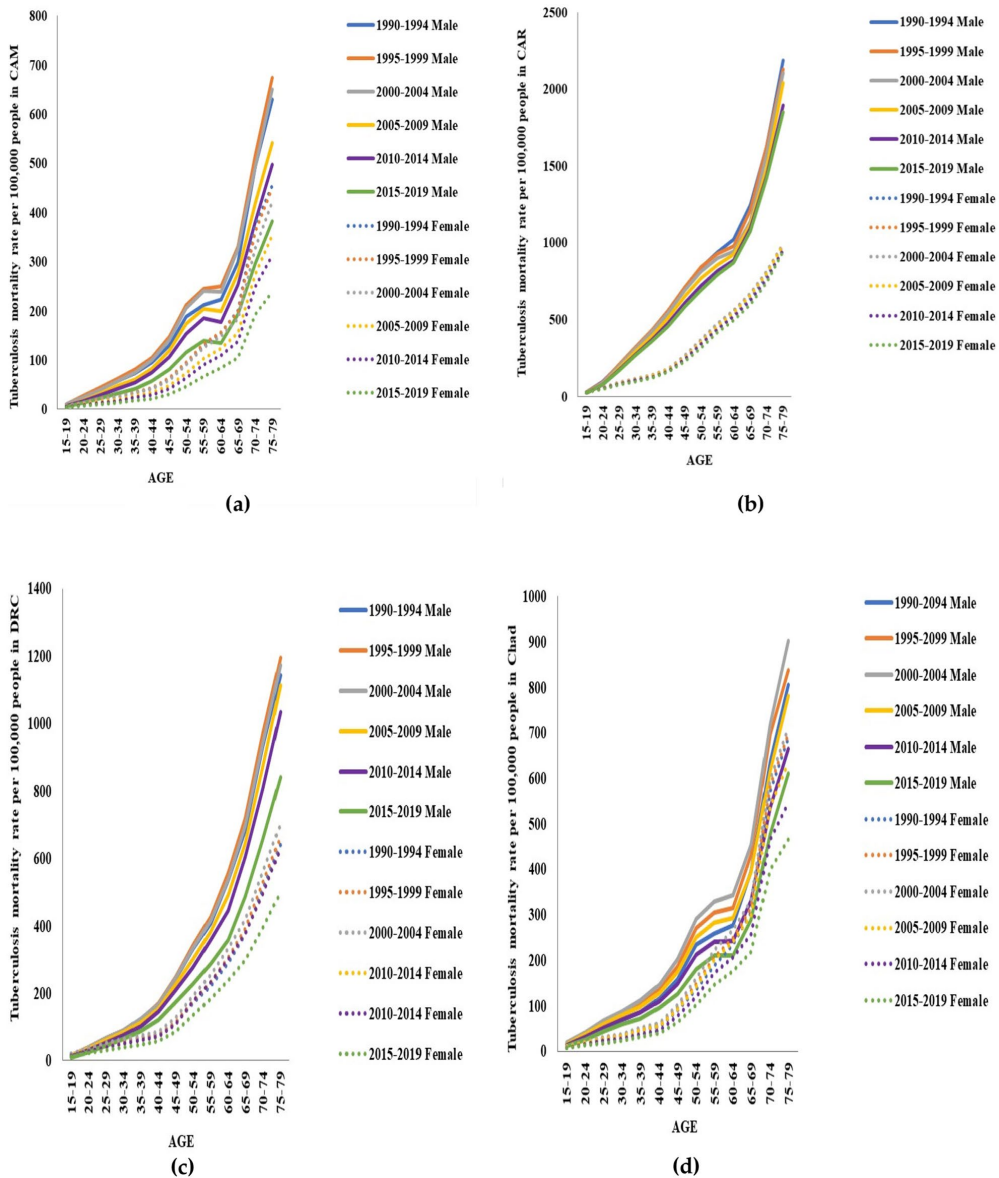
Tuberculosis mortality rates in each age group in males and females from Cameroon (CAM) (**a**), Central African Republic (CAR) (**b**), the Democratic Republic of the Congo (DRC) (**c**), and Chad (**d**) during 1990–2019.

Figure 4 showed that TB mortality rates in males and females of the four countries exponentially increased with age. The lowest rates were in the age group of 15–19 years, and the peak points were in individuals aged between 75 and 79 years. In both genders, TB mortality rates in different age groups were low during 2015–2019, and the rates in the male gender were higher compared to the female gender in all four countries. TB mortality rates also displayed similar patterns in the trends in males and females from CAM and Chad, and CAR and DRC respectively.

The age relative risks (RRs) of TB incidence and mortality in men and women of CAM, CAR, DRC, and Chad are represented in Tables 3, and 4. Relatively to the age RRs, men and women aged between 15 and 54 years are at high risk of TB incidence in the four countries (Table 3). Males and females aged between 15 and 49 of the four countries are at high risk of TB mortality based on the age RRs (Table 4).

**Table 4:** Results of the Age-Period-Cohort model analysis of Tuberculosis incidence in males and females from Cameroon (CAM), Central African Republic (CAR), the Democratic Republic of the Congo (DRC), and Chad between 1990 and 2019.

Variables	CAM		CAR		DRC		Chad	
	Males RR	Females RR	Males RR	Females RR	Males RR	Females RR	Males RR	Females RR
**Age**								
15–19	2.50	4.03	1.85	2.60	2.13	2.53	1.76	2.83
20–24	2.15^↓^	3.21^↓^	1.66^↓^	2.20^↓^	1.90^↓^	2.16^↓^	1.6^↓^	2.44^↓^
25–29	1.84^↓^	2.56^↓^	1.50^↓^	1.86^↓^	1.69^↓^	1.84^↓^	1.45^↓^	2.11^↓^
30–34	1.58^↓^	2.03^↓^	1.35^↓^	1.58^↓^	1.51^↓^	1.57^↓^	1.32^↓^	1.82^↓^
35–39	1.36^↓^	1.62^↓^	1.21^↓^	1.34^↓^	1.34^↓^	1.34^↓^	1.20^↓^	1.57^↓^
40–44	1.17^↓^	1.29^↓^	1.09^↓^	1.13^↓^	1.19^↓^	1.14^↓^	1.09^↓^	1.36^↓^
45–49	1.00^↓^	1.03^↓^	0.98^↓^	0.96^↓^	1.06^↓^	0.97^↓^	0.99^↓^	1.17^↓^
50–54	0.86^↓^	0.82^↓^	0.88^↓^	0.81^↓^	0.94^↓^	0.83^↓^	0.90^↓^	1.01^↓^
55–55	0.74^↓^	0.65^↓^	0.80^↓^	0.69^↓^	0.84^↓^	0.71^↓^	0.81^↓^	0.87^↓^
60–64	0.63^↓^	0.52^↓^	0.72^↓^	0.58^↓^	0.75^↓^	0.60^↓^	0.74^↓^	0.76^↓^
65–69	0.54^↓^	0.41^↓^	0.64^↓^	0.49^↓^	0.66^↓^	0.51^↓^	0.67^↓^	0.65^↓^
70–74	0.47^↓^	0.33^↓^	0.58^↓^	0.42^↓^	0.59^↓^	0.44^↓^	0.61^↓^	0.56^↓^
75–79	0.40^↓^	0.26^↓^	0.52^↓^	0.35^↓^	0.53^↓^	0.37^↓^	0.55^↓^	0.49^↓^
**Period**								
1990–1994	1.27	1.33	1.21	1.04	1.20	1.28	1.16	1.26
1995–1999	1.12^↓^	1.15^↓^	1.13^↓^	1.06^↑^	1.10^↓^	1.13^↓^	1.05^↓^	1.09^↓^
2000–2004	1^↓^	1^↓^	1^↓^	1^↓^	1^↓^	1^↓^	1^↓^	1^↓^
2005–2009	0.85^↓^	0.81^↓^	0.85^↓^	0.84^↓^	0.88^↓^	0.86^↓^	0.91^↓^	0.89^↓^
2010–2014	0.73^↓^	0.61^↓^	0.80^↓^	0.64^↓^	0.78^↓^	0.71^↓^	0.82^↓^	0.74^↓^
2015–2019	0.58^↓^	0.413^↓^	0.73^↓^	0.45^↓^	0.66^↓^	0.57^↓^	0.70^↓^	0.58^↓^
**Cohort**								
1915–1919	3.05	4.66	3.22	3.25	2.60	5.21	2.26	1.97
1920–1924	2.75^↓^	4.12^↓^	2.88^↓^	3.72^↑^	2.59^↓^	5.05^↓^	1.97^↓^	1.81^↓^
1925–1929	2.51^↓^	3.48^↓^	2.09^↓^	3.16^↓^	1.94^↓^	3.46^↓^	1.70^↓^	1.63^↓^
1930–1934	2.16^↓^	2.74^↓^	1.46^↓^	2.01^↓^	1.44^↓^	2.12^↓^	1.49^↓^	1.50^↓^
1935–1939	1.81^↓^	2.16^↓^	1.44^↓^	1.65^↓^	1.29^↓^	1.59^↓^	1.37^↓^	1.38^↓^
1940–1944	1.45^↓^	1.68^↓^	1.32^↓^	1.44^↓^	1.22^↓^	1.34^↓^	1.39^↑^	1.29^↓^
1945–1949	1.23^↓^	1.37^↓^	1.22^↓^	1.27^↓^	1.11^↓^	1.15^↓^	1.28^↓^	1.21^↓^
1950–1954	1.12^↓^	1.16^↓^	1.12^↓^	1.10^↓^	1.038^↓^	1.04^↓^	1.13^↓^	1.10^↓^
1955–1959	1^↓^	1^↓^	1^↓^	1^↓^	1^↓^	1^↓^	1^↓^	1^↓^
1960–1964	0.82^↓^	0.82^↓^	0.85^↓^	0.81^↓^	0.91^↓^	0.91^↓^	0.81^↓^	0.82^↓^
1965–1969	0.66^↓^	0.62^↓^	0.73^↓^	0.54^↓^	0.78^↓^	0.76^↓^	0.70^↓^	0.63^↓^
1970–1974	0.57^↓^	0.46^↓^	0.69^↓^	0.43^↓^	0.61^↓^	0.58^↓^	0.67^↓^	0.51^↓^
1975–1979	0.54^↓^	0.34^↓^	0.66^↓^	0.39^↓^	0.55^↓^	0.49^↓^	0.72^↑^	0.44^↓^
1980–1984	0.52^↓^	0.26^↓^	0.64^↓^	0.41^↑^	0.50^↓^	0.44^↓^	0.70^↓^	0.38^↓^
1985–1989	0.43^↓^	0.22^↓^	0.60^↓^	0.38^↓^	0.46^↓^	0.41^↓^	0.62^↓^	0.32^↓^
1990–1994	0.32^↓^	0.17^↓^	0.52^↓^	0.36^↓^	0.41^↓^	0.36^↓^	0.50^↓^	0.26^↓^
1995–1999	0.24^↓^	0.13^↓^	0.43^↓^	0.31^↓^	0.35^↓^	0.30^↓^	0.40^↓^	0.20^↓^
2000–2004	0.19^↓^	0.10^↓^	0.37^↓^	0.25^↓^	0.28^↓^	0.25^↓^	0.33^↓^	0.16^↓^
**Wald tests**								
Age RR	p ˂ 0.01							
Period RR	p ˂ 0.01							
Cohort RR	p ˂ 0.01							

*RR* relative risks, *P* alpha value; ^↑^, upward values; ^↓^, downward values.

### The period relative risks of TB incidence and mortality by gender in Cameroon, Chad, Central African Republic, and the Democratic Republic of the Congo

Period relative risks or period risk ratio (RRs) of TB incidence in CAM, CAR, DRC, and Chad have decreased from 1990 to 2019 in both males and females, except for the females in CAR which had an upward behavior of nearly 50% from 1997.5 (median 1995–1999) before declining until 2019. According to the gender, the period RRs of TB incidence decreased in males by almost 2.2, 1.6, 1.8, and 1.7 times in CAM, CAR, DRC, and Chad respectively. It has decreased by almost 3.2, 2.3, 2.2, and 2.2 folds in females of the four countries accordingly as displayed in Table 4. Based on their respective values in each gender, TB incidence period RRs were very high in males and females from CAM during 1990–1994. Between the same period (1990–1994), men from Chad and women from CAR had the lowest TB incidence period RRs compared to others. Between 2015 and 2019, men and women from CAM had the lowest values of period RRs, and males from Chad and females from DRC were found to have the highest TB incidence period RRs compared to males and females from the rest of the countries.

Table 4 displayed the period relative risks (RRs) of TB mortality in men and women of the four countries. During the period of 1997.5 (median 1995–1999), period RRs of TB mortality increased marginally by 50% and 51% in males from CAM and Chad respectively. In 2002.5 (median 2000–2004), females from DRC had upward behavior of the period RRs which was around 50% compared to the previous period (1995–1999). From 1990 to 2019, period RRs of TB mortality had decreased by nearly 3.3 times in men from CAM. The decrease of TB mortality in the females from CAM was dramatically around 3.7 folds. In CAR, period RRs of TB mortality had suddenly decreased by almost 1.9 times in males, and 1.7 times in females between 1990 and 2019. DRC males and females had period RRs which also declined dramatically by nearly 2.7 and 2 times alternatively. Chad had witnessed the declining behavior of period RRs by approximately 2.2 times in men, and 2.3 folds in women from 1990 to 2019. From 1990 through 1994, the period RRs of TB mortality in males from DRC and females from CAM was the highest. Males from Chad and females from DRC had instead the lowest period RRs values compared to others. Across 2015–2019, men and women from CAR had the highest, and those from CAM had the lowest period RRs of TB mortality compared to the rest of men and women from the other countries.

### The cohort relative risks of TB incidence and mortality by gender in Cameroon, Chad, the Central African Republic, and the Democratic Republic of the Congo

The cohort relative risks or risk ratios (RRs) of TB incidence declined in CAM, CAR, DRC, and Chad in both men and women (Table 4). It decreased accordingly by nearly 16 and 9 times in males, and almost 47 and 21 times in females from CAM and DRC, respectively. A dramatic decrease could also be noticed in the cohort RRs of men from CAR which was around 8.6 times. In women of the same country (CAR) the cohort RRs decreased by nearly 13 folds, although the cohort born in 1920–1924 and 1980–1984 had respectively raised by nearly 53% and 51% compared to their respective previous cohort groups (1915–1919 and 1975–1979). The cohort RRs of TB incidence in Chadian males have also slightly increased by almost 50% and 52% in those born between 1940–1944 and 1975–1979 respectively. The cohort RRs of TB incidence had decreased around 6.7- and 12-folds in Chadian males and females respectively, born between 1915 and 2004. In the cohort born in 1915–1919, males from CAR and females from DRC had the highest values compared to others. The cohort RRs of TB incidence decreased the most in men and women born in 2000–2004 from CAM compared to the rest of the countries as displayed in Table 4.

In Table 5, the cohort relative risks (RRs) of TB mortality in both men and women had decreased in all four countries, and the cohort RRs of men and women from CAM born in 2000–2004 were approximately equal (0.07). It had decreased rapidly around 70- to 97-folds in males and females from CAM respectively. In the cohort born in 1920–1924, women from CAR and DRC had seen their cohort RRs slightly increasing (50% and 54% accordingly) relative to those born in 1915–1919. Males from CAR born in 1935–1939 and those from Chad born in 1940–1944 also showed similar behavior with a nearly 50% increase in CAR and Chad. In CAR, the decrease was almost 15.1 times in males and around 6.4 times in females from the early cohort group to the last. DRC also showed a rapid decrease of cohort RRs in the male gender by approximately 35.7-folds in those born from 1915 to 2004. The cohort RRs of TB mortality in DRC females declined by nearly 13 times. Chadian men and women born between 1915 and 2004 also had met a decreasing pattern in the cohort RRs associated with TB mortality. As a result, the cohort RRs of TB mortality had declined by almost 24- and 27.2-folds accordingly in Chadian men and women. TB mortality cohort RRs decreased the most in males and females from CAM, although they were the highest in the cohort born in 1915–1919 compared to the rest of the men and women from CAR, DRC, and Chad.

**Table 5:** Results of the Age-Period-Cohort model analysis of Tuberculosis mortality in males and females from Cameroon (CAM), Central African Republic (CAR), the Democratic Republic of the Congo (DRC), and Chad between 1990 and 2019.

Variables	CAM		CAR		DRC		Chad	
	Males RR	Females RR	Males RR	Females RR	Males RR	Females RR	Males RR	Females RR
**Age**								
15–19	3.99	4.30	2.59	1.75	3.64	2.13	2.62	2.85
20–24	3.12^↓^	3.31^↓^	2.26^↓^	1.58^↓^	2.99^↓^	1.86^↓^	2.20^↓^	2.39^↓^
25–29	2.45^↓^	2.54^↓^	1.97^↓^	1.42^↓^	2.45^↓^	1.62^↓^	1.85^↓^	2.01^↓↓^
30–34	1.92^↓^	1.95^↓^	1.71^↓^	1.28^↓^	2.00^↓^	1.41^↓^	1.55^↓^	1.69^↓^
35–39	1.50^↓^	1.50^↓^	1.49^↓^	1.15^↓^	1.64^↓^	1.23^↓^	1.31^↓^	1.42^↓^
40–44	1.18^↓^	1.15^↓^	1.30^↓^	1.04^↓^	1.34^↓^	1.07^↓^	1.10^↓^	1.20^↓^
45–49	0.92^↓^	0.89^↓^	1.13^↓^	0.94^↓^	1.10^↓^	0.93^↓^	0.92^↓^	1.01^↓^
50–54	0.72^↓^	0.68^↓^	0.99^↓^	0.84^↓^	0.90^↓^	0.81^↓^	0.77^↓^	0.84^↓^
55–59	0.57^↓^	0.52^↓^	0.86^↓^	0.76^↓^	0.74^↓^	0.71^↓^	0.65^↓^	0.71^↓^
60–64	0.44^↓^	0.40^↓^	0.75^↓^	0.68^↓^	0.60^↓^	0.61^↓^	0.55^↓^	0.60^↓^
65–69	0.35^↓^	0.31^↓^	0.65^↓^	0.62^↓^	0.49^↓^	0.53^↓^	0.46^↓^	0.50^↓^
70–74	0.27^↓^	0.23^↓^	0.57^↓^	0.55^↓^	0.40^↓^	0.47^↓^	0.38^↓^	0.42^↓^
75–79	0.21^↓^	0.18^↓^	0.49^↓^	0.50^↓^	0.33^↓^	0.41^↓^	0.32^↓^	0.35^↓^
**Period**								
1990–1994	1.12	1.23	1.23	1.06	1.27	1.02	0.99	1.08
1995–1999	1.14^↓^	1.16^↓^	1.17^↓^	1.04^↓^	1.17^↓^	0.98^↓^	1.03^↓^	1.05^↓^
2000–2004	1^↓^	1^↓^	1^↓^	1^↓^	1^↓^	1^↑^	1^↓^	1^↓^
2005–2009	0.72^↓^	0.72^↓^	0.77^↓^	0.89^↓^	0.80^↓^	0.91^↓^	0.76^↓^	0.79^↓^
2010–2014	0.54^↓^	0.53^↓^	0.74^↓^	0.74^↓^	0.65^↓^	0.70^↓^	0.57^↓^	0.60^↓^
2015–2019	0.34^↓^	0.33^↓^	0.65^↓^	0.63^↓^	0.46^↓^	0.48^↓^	0.44^↓^	0.47^↓^
**Cohort**								
1915–1919	5.54	7.13	3.02	2.23	4.08	2.69	3.75	3.46
1920–1924	4.40^↓^	5.98^↓^	2.58^↓^	2.68^↑^	3.77^↓^	3.14^↑^	2.81^↓^	2.64^↓^
1925–1929	3.60^↓^	4.74^↓^	1.83^↓^	2.31^↓^	2.66^↓^	2.60^↓^	2.25^↓^	2.05^↓^
1930–1934	2.87^↓^	3.53^↓^	1.24^↓^	1.53^↓^	1.89^↓^	1.65^↓^	1.81^↓^	1.68^↓^
1935–1939	2.32^↓^	2.67^↓^	1.28^↑^	1.31^↓^	1.60^↓^	1.39^↓^	1.60^↓^	1.50^↓^
1940–1944	1.80^↓^	2.02^↓^	1.19^↓^	1.21^↓^	1.43^↓^	1.24^↓^	1.61^↑^	1.44^↓^
1945–1949	1.42^↓^	1.54^↓^	1.08^↓^	1.09^↓^	1.24^↓^	1.11^↓^	1.39^↓^	1.29^↓^
1950–1954	1.19^↓^	1.22^↓^	1.03^↓^	1.00^↓^	1.09^↓^	1.01^↓^	1.14^↓^	1.12^↓^
1955–1959	1^↓^	1^↓^	1^↓^	1^↓^	1^↓^	1^↓^	1^↓^	1^↓^
1960–1964	0.81^↓^	0.81^↓^	0.90^↓^	0.99^↓^	0.85^↓^	0.96^↓^	0.81^↓^	0.83^↓^
1965–1969	0.64^↓^	0.64^↓^	0.77^↓^	0.92^↓^	0.67^↓^	0.86^↓^	0.65^↓^	0.69^↓^
1970–974	0.48^↓^	0.48^↓^	0.61^↓^	0.79^↓^	0.47^↓^	0.71^↓^	0.52^↓^	0.54^↓^
1975–1979	0.35^↓^	0.35^↓^	0.48^↓^	0.67^↓^	0.36^↓^	0.57^↓^	0.44^↓^	0.44^↓^
1980–1984	0.26^↓^	0.26^↓^	0.39^↓^	0.58^↓^	0.29^↓^	0.46^↓^	0.36^↓^	0.35^↓^
1985–1989	0.19^↓^	0.18^↓^	0.33^↓^	0.50^↓^	0.23^↓^	0.38^↓^	0.31^↓^	0.28^↓^
1990–1994	0.13^↓^	0.13^↓^	0.28^↓^	0.44^↓^	0.18^↓^	0.31^↓^	0.25^↓^	0.21^↓^
1995–1999	0.10^↓^	0.10^↓^	0.23^↓^	0.39^↓^	0.14^↓^	0.26^↓^	0.20^↓^	0.16^↓^
2000–2004	0.07^↓^	0.07^↓^	0.19^↓^	0.34^↓^	0.11^↓^	0.20^↓^	0.15^↓^	0.12^↓^
**Wald tests**								
Age RR	p ˂ 0.01							
Period RR	p ˂ 0.01							
Cohort RR	p ˂ 0.01							

*RR* relative risks, *p* alpha value; ^↑^, upward values; ^↓^, downward values.

The Wald tests demonstrated a statistical significance of the local drifts, net drifts, age, period, and cohort RRs at alpha p ˂ 0.01.

## Discussion

Our study demonstrated that the trends of tuberculosis (TB) incidence and mortality have both decreased in males and females from Cameroon (CAM), the Central African Republic (CAR), the Democratic Republic of the Congo (DRC), and Chad; except for the males of DRC which displayed an almost-steady pattern in the trend of TB incidence from 1990 to 2019. TB incidence and mortality patterns in males were higher compared to females of the four countries. CAR recorded the highest and CAM the lowest TB ASIR and ASMR in males and females compared to the rest of the countries. According to the Joinpoint regression analysis, TB ASIR and ASMR have significantly decreased the most in males and females from CAM; meanwhile, males and females from DRC and CAR had the lowest decrease compared to the rest of the countries between 1990 and 2019. The association of TB with socio-economic factors such as poverty and tobacco smoking and alcohol use disorder was demonstrated in many previous studies^2,4^. Poverty alleviation and promotion of a better lifestyle and behavior are some of the risk factors that needed to be solved to reach the TB target set by the SDGs^18^. When observing the trends of the human development index (HDI) that represents a better socio-economic status, education level, and living conditions in the populations^8^, CAM had a continuously increasing trend compared to the rest of the countries^9^. Therefore, the most significant decrease in the trends of TB ASIR and ASMR in males and females from CAM might be induced by this continuous growth of the HDI between 1990 and 2019 that proved the efforts and improvements made to positively change the socio-economic status, the educative status, including better living conditions of the men and women from CAM. Oppositely, the trends of the HDI in DRC and CAR showed increasing behavior only from 2000–2019 and 2010–2019 respectively^9^. This factor might be behind the fact TB ASIR and ASMR decreased less in the males and females from DRC and CAR respectively. Furthermore, the high association of the male gender to TB ASIR and ASMR compared to the female gender might be the result of the fact that women's socio-economic rights values evaluated to be low between zero to one (0–1) in the four countries^25^. This could mean that men are the bread-earners in societies, making them more exposed to TB incidence and mortality risk factors during their daily activities. When observing the trends of TB incidence and mortality in males and females of the four countries, the decrease was not rapid over our study period according to the results of the Joinpoint regression analysis. This slow decrease of TB incidence and mortality in the four countries might not positively affect the marathon towards the “End TB pandemic strategy” of the World Health Organization (WHO). The governments of CAM, CAR, DRC, and Chad should imply new policies in the same direction as the SDGs set by the United Nations (UN) to impact positively the fight against the TB burden in men and women. Social protection and ending extreme poverty in respective populations should be the nucleus of those new policies^5,18^.

In our study, we used an Age-Period-Cohort (APC) model analysis to dissect the contribution of age, period, and birth cohort as relative risks to TB incidence and mortality in the men and women from CAM, CAR, DRC, and Chad. We will analyze the different factors that impacted each pattern in the following parts of our study to provide a comprehensive analysis of TB incidence and mortality in males and females of those four countries.

The APC model used to examine the association of age, period, and birth cohort as relative risks to TB incidence and mortality generated the following outcomes. Based on the local drifts (annual percentage of changes in each age group from 15–19 to 75–79 years), TB incidence and mortality rates in different age groups uncovered sinusoidal patterns with upward and downward behavior which involved almost every age group in men and women from the four countries. In Chad, the upward behavior of TB incidence rates started in females aged from 50 years old. The overall annual percentage changes over time for the adjusted age group (15–79 years old) represented by the net drift demonstrated that TB incidence and mortality rates in males and females from CAM considerably decreased the most compared to men and women from the rest of the countries. TB incidence and mortality rates respectively decreased less in males and females from Chad and CAR according to the values of their net drifts.

Relatively to age, TB incidence and mortality rates increased from the age group of 15–19 to 75–79 years old in males and females of the four countries from 1990 to 2019. As age increases, numerous medical conditions such as diabetes affect individuals and their immune system becomes weaker^26^. When the immune system becomes weaker, vulnerability increases, and conditions such as malnutrition, severe kidney diseases, and indoor air pollution might affect the health of elderlies rending them more vulnerable to other infections and diseases^26,27^. The association of these factors in elderlies with TB incidence and mortality has been analyzed in many previous studies^28–30^. The age relative risks (RRs) outlined that the men and women aged between 15 and 54 years old have high risks of TB incidence, and those aged between 15 and 49 years old are at high risk of TB mortality in the four countries. Factors such as the human immunodeficient virus (HIV) infection, smoking, and heavy alcohol consumption (unhealthy lifestyle) might be the major causes of this finding^31–33^. An unhealthy lifestyle is usually linked to young adults and middle-aged adults age categories, and many studies have found them to be directly associated with TB incidence and mortality^34–37^. Furthermore, the HIV prevalence in new and relapse TB cases per age group showed that in all four countries, the men and women aged between 10 and 49 years were associated with HIV new and relapse TB cases^5^. This finding might be the reason for the involvement of the age categories of 15–54 and 15–49 years old with TB incidence and mortality respectively in the four countries. Stronger public health policies against smoking (especially in a public environment) and alcohol drinking might help alleviate the burden of TB in both genders across the four countries^36^. Moreover, new health strategies and approaches towards adults and elderlies in both genders, improvement of TB control infrastructures in rural and urban areas, promotion of better living standards, poverty alleviation, and massive TB screening campaigns for men and women should be untaken seriously by respective governments of those countries to strengthen TB control. Particular attention and emphasis should be considered by policymakers for the elderlies. Promotion of healthy lifestyle especially in young adults and adults, improvement of the health conditions in elderlies should be improved to reduce TB co-morbidity such as diabetes and HIV infection^37,38^. Such approaches might considerably reduce the age relative risks associated with TB incidence and mortality in both genders of the four countries.

The period RRs on TB incidence and mortality had decreased in both genders of all four countries between 1990 and 2019. The decrease of TB incidence and mortality period relative risks (RRs) might be associated with the efforts made by respective governments in declining TB burden on their populations through the settings of national TB control programs and organizations in CAM, CAR, DRC, and Chad^39–42^. Besides these remarkable efforts, there were some upward behaviors in the period RRs in some countries over few periods. These upward changes might be the result of the impact of socio-economic and political factors. Firstly, all four countries have very low human development index values during our study period (1990–2019)^9^. This reflects the low living condition of the populations in terms of the healthcare system, education, and socio-economic^8^. Secondly, the upward behavior of the period RRs might be directly correlated to the trends of the gross domestic product annual rate of each country. During the periods when the period RRs had upward behaviors, CAR, CAM, DRC, and Chad displayed consequently a decreasing behavior in their respective gross domestic product (GDP) annual growth^43–46^. The decrease of the GDP might have affected the ability of the respective governments to provide a better and healthy lifestyle to the population, efficient healthcare strategies and structures, and alleviation of poverty. In DRC, the upward behavior of the TB mortality period RRs in women during 2000–2004 might be caused by medical conditions that weaken the immune system during pregnancy in women living with TB and HIV infection^47^. HIV prevalence in women from DRC was found to be higher than males^48^. Therefore, a particular focus on the socio-economic status and women's health might contribute to decreasing the TB burden in males and females of the four countries.

The cohort RRs of TB incidence and mortality had both substantially decreased in all four countries in males and females born from 1915 to 2004, although some upward behaviors were observed in some birth cohort groups across the countries except for males and females from CAM. Cohort RRs are very necessary, and major variations of the cohort RRs indicate the balance between weakened immune system responses from previous and new infections^49^. Because period effects can have an impact on certain age categories causing an indirect cohort effect, and people from various cohorts born in separate periods can cause uncertainty about the period association in some ways, it is relatively difficult to decompose the interpretation of those models in the real-world^24^. As a result, the changes in the cohort RRs of TB incidence and mortality in males and females of the four countries are associated with the different changes from different periods in each of the countries. The governments of CAM, CAR, DRC, and Chad should imply new approaches and policies to heavily impact the decrease of TB incidence and mortality in the male and female population. Governmental and non-governmental organizations might need to multiply diagnosis strategies and structures for massive campaigns of TB screening and education. Conceptualization and realization of special infrastructures to accommodate patients diagnosed with TB and thus, enhance TB control might need to be considered. An implementation of a regional TB control program might be directly beneficial to decreasing the burden of TB in those countries (CAM, CAR, DRC, and Chad). Many of the problems encountered by the countries are resemblant due to their similar socio-economic, socio-cultural, socio-politic, and geographical factors^6,7,9^. Consequently, particular attention should be given by each government to alleviate poverty, to ameliorate women's health and ensure gender equity, and to provide better living standards. Such an agenda might help to better address TB incidence and mortality in males and females of the four countries. Special interventions and programs should be launched for elderlies who smoke and with TB comorbidities^36–38^. Because the burden of TB is centralized in low-income and middle-income countries, the successful implementation of social protection and extreme poverty eradication might significantly impact the decrease of TB incidence and mortality in men and women from CAM, CAR, DRC, and Chad. Although the trend of multi-drug resistant tuberculosis (MDR TB) displayed a low prevalence of MDR TB estimates in Sub-Saharan Africa^50^, With the outbreak of the novel coronavirus (Covid-19), many countries had to lock down their borders with quarantine and travel restrictions which induced economic crisis and psychological changes^51^. Such unpredictable variables might slow down the accomplishment of the “End TB pandemic strategy”; thus, influencing the achievement of the SDGs^5,18^. Because of the regional and the national level of the fight against TB, respective governments should instore TB check-point agencies at borders and airports to check for TB test results, TB vaccination certificates, and/or TB treatment medical records in the populations moving within the four countries as seen in the case of Covid-19^51^. This might help to control the spread of TB, and strengthen the management and treatment of TB over the countries of the region.

There were some limitations in our study. Firstly, this article does not analyze different subgroups of tuberculosis. Secondly, the other constraint is related to the Age-Period-Cohort analysis (identifiability and uncertainty principle). These parameters could not be avoided because the interpretation of results from an individual’s level does not necessarily equate to the interpretation at the population level. However, many other studies were conducted using the web tool APC model as we did in our study^52,53^. Therefore, large-scale and TB subgroups studies should be performed in the future to validate the hypothesis associated with our study.

## Conclusions

Our study demonstrated that although slow, tuberculosis incidence and mortality have decreased in males and females from Cameroon, the Central African Republic, the Democratic Republic of the Congo, and Chad from 1990 to 2019, and the most significant decrease was noticed in Cameroon. The male gender was found to be more associated with TB incidence than TB mortality, compared to the female gender of all four countries. Males and females aged between 15 and 54 years are at high risk of TB incidence, and those aged between 15 and 49 years of TB mortality. To reach the goal set by the world health organization (WHO) to “End Tuberculosis pandemic” by decreasing substantially Tuberculosis burden, TB incidence, and mortality in men and women from Cameroon, Chad, Central African Republic, and the Democratic Republic of the Congo should decrease rapidly compared to their actual trends. Governmental and non-governmental organizations should strengthen policies against TB incidence in the male gender of the four countries. The female gender should be educated about TB mortality risks. Poverty alleviation in both genders of the four countries might provide positive outcomes in the fight against the burden of TB in those countries. Furthermore, the health conditions of elderlies should be ameliorated to decrease TB comorbidities in their age category.

## Data Availability

The datasets analyzed during this study are publicly available in the Global Burden of Disease (GBD) repository, available from: http://ghdx.healthdata.org/gbd-results-tool^24^.

## Abbreviations

APCM: Age-period-cohort model
APC: Annual percentage change
AAPC: Average annual percent change
ASIR: Age-standardized incidence rate
ASMR: Age-standardized mortality rate
CAM: Cameroon
CAR: The Central African Republic
CI: Confidence interval
CIR: Crude incidence rate
CMR: Crude mortality rate
Covid-19: The novel coronavirus
DRC: The Democratic Republic of the Congo
GHDx: Global Health Data exchange
GBD: Global Burden of Disease
HDI: Human Development Index
HIV: Human immunodeficiency virus
IHME: Institute of Health Metrics and Evaluation
RRs: Relative risks
SDGs: Sustainable development goals
TB: Tuberculosis
UN: United Nations
UNDP: United Nations Development Programme
WHO: World Health Organization

## References

[1] Koch A, Mizrahi V (2018). *Mycobacterium tuberculosis*. Trends Microbiol..

[2] World Health Organization. Tuberculosis. (2020). https://www.who.int/news-room/fact-sheets/detail/tuberculosis (Accessed Nov 2020).

[3] Centers for Disease Control and Prevention. Tuberculosis. (2020). https://www.cdc.gov/globalhealth/newsroom/topics/tb/ (Accessed Nov 2020).

[4] Zaman K (2010). Tuberculosis: A global health problem. J. Health Popul. Nutr..

[5] World Health Organization. Global Tuberculosis Report 2020. (2020). https://www.who.int/publications/i/item/9789240013131. (Accessed June 2020).

[6] The University of Pittsburgh. Central African Countries. (2020). https://pitt.libguides.com/c.php?g=12378&p=65816. (Accessed Nov 2020).

[7] Hannah Ritchie and Max Roser. "Gender Ratio". Published online at OurWorldInData.org. (2019). https://ourworldindata.org/gender-ratio. (Accessed Nov 2020).

[8] United Nations Development Programme. Human Development Index (HDI) Ranking. 2020 Human Development Report. http://hdr.undp.org/en/content/latest-human-development-index-ranking. (Accessed Dec 2020).

[9] United Nations United Nations Development Programme. *Human Development Index*. (2019). http://hdr.undp.org/sites/default/files/2020_statistical_annex_table_2.pdf. (Accessed June 2021).

[10] KNOEMA. Cameroon—Tuberculosis death rate (cases per 100,000 people). (2020). https://knoema.com/atlas/Cameroon/topics/Health/Risk-factors/Tuberculosis-death-rate?compareTo=TD,CD,CF. (Accessed Jan 2021).

[11] Ngangro NN, Ngarhounoum D, Ngangro MN, Rangar N, Siriwardana MG, Des Fontaines VH, Chauvin P (2012). Pulmonary tuberculosis diagnostic delays in Chad: A multicenter, hospital-based survey in Ndjamena and Moundou. BMC Public Health.

[12] Noeske J, Ndi NF, Amougou Elo G, Mbondi Mfondih S (2014). Tuberculosis incidence in Cameroonian prisons: A 1-year prospective study. S. Afr. Med. J..

[13] Tanue EA, Nsagha DS, Njamen TN, Clement Assob NJ (2019). Tuberculosis treatment outcome and its associated factors among people living with HIV and AIDS in Fako Division of Cameroon. PLoS One.

[14] Nouvel LX, Kassa-Kelembho E, Dos Vultos T, Zandanga G, Rauzier J, Lafoz C, Martin C, Blazquez J, Talarmin A, Gicquel B (2006). Multidrug-resistant *Mycobacterium tuberculosis*, Bangui, Central African Republic. Emerg. Infect. Dis..

[15] Janssen S, Huson MAM, Bélard S, Stolp S, Kapata N, Bates M, Van Vugt M, Grobusch MP (2014). TB and HIV in the Central African region: Current knowledge and knowledge gaps. Infection.

[16] Kayomo MK, Hasker E, Aloni M, Nkuku L, Kazadi M, Kabengele T, Muteteke D, Kapita F, Lufulwabo A, Mukadi YD, Muyembe-Tamfum JJ, Ieven M, de Jong BC, Boelaert M (2018). Outbreak of tuberculosis and multidrug-resistant tuberculosis, Mbuji-Mayi Central Prison, Democratic Republic of the Congo. Emerg. Infect. Dis..

[17] Diguimbaye, C. La tuberculose humaine et animale au Tchad: contribution à la mise en évidence et caractérisation des agents causaux et leur implication en santé publique. (2004). https://edoc.unibas.ch/219/1/DissB_7142.pdf. Accessed Feb 2021.

[18] Menzies Carter DJ, Glaziou P, Lönnroth K, Siroka A, Floyd K, Weil D, Raviglione M, Houben R, Boccia D (2018). The impact of social protection and poverty elimination on global tuberculosis incidence: A statistical modeling analysis of Sustainable Development Goal 1. Lancet Glob. Health.

[19] Global Burden of Disease Collaborative Network. Global Burden of Disease Study 2019 (GBD 2019) Results. Seattle, United States: Institute for Health Metrics and Evaluation (IHME), 2020. http://ghdx.healthdata.org/gbd-results-tool. (Accessed Dec 2020).

[20] Zou Z, Cini K, Dong B, Ma Y, Ma J, Burgner DP, Patton GC (2020). Time trends in cardiovascular disease mortality across the BRICS: An age-period-cohort analysis of key nations with emerging economies using the global burden of disease study. Circulation.

[21] Holford TR (1983). The estimation of age, period and cohort effects for vital rates. Biometrics.

[22] Robertson C, Gandini S, Boyle P (1999). Age-period-cohort models: A comparative study of available methodologies. J. Clin. Epidemiol..

[23] Rosenberg PS, Anderson WF (2012). Age-period-cohort models in cancer surveillance research: Ready for prime time?. Cancer Epidemiol. Biomark. Prev..

[24] Holford, T. Age-period-cohort analysis. In *Wiley StatsRef: Statistics Reference Online* (Wiley, 2005).

[25] Esteban, O.-O. & Max, R. Economic inequality by gender. Published online at OurWorldInData.org. (2018). https://ourworldindata.org/economic-inequality-by-gender. (Accessed Feb 2021).

[26] Stevenson CR, Forouhi NG, Roglic G, Williams BG, Lauer JA, Dye C, Unwin N (2007). Diabetes and tuberculosis: The impact of the diabetes epidemic on tuberculosis incidence. BMC Public Health.

[27] Thomas TY, Rajagopalan S (2001). Tuberculosis and aging: A global health problem. Clin. Infect. Dis..

[28] Moutschen M, Scheen A, Lefebvre P (1992). Impaired immune responses in diabetes mellitus: Analysis of the factors and mechanisms involved, relevance to the increased susceptibility of diabetic patients to specific infections. Diabetes Metab..

[29] Cegielski J, McMurray D (2004). The relationship between malnutrition and tuberculosis: Evidence from studies in humans and experimental animals. Int. J. Tuberc. Lung Dis..

[30] Centers for Disease Control and Prevention. TB Risk Factors. (2016). https://www.cdc.gov/tb/topic/basics/risk.htm. (Accessed Feb 2021).

[31] NIH’s Office of Aids Research. HIV and Tuberculosis (TB). HIV and Opportunistic Infections, Coinfections, and Conditions. (2020). https://hivinfo.nih.gov/understanding-hiv/fact-sheets/hiv-and-tuberculosis-tb#. (Accessed Feb 2021).

[32] Den Boon S, Van Lill SWP, Borgdorff MW, Verver S, Bateman ED, Lombard CJ, Enarson DA, Beyers N (2005). Association between smoking and tuberculosis infection: A population survey in a high tuberculosis incidence area. Thorax.

[33] Cui Y, Shen H, Wang F, Wen H, Zeng Z, Wang Y (2005). A long-term trend study of tuberculosis incidence in China, India and United States 1992–2017: A joinpoint and age-period-cohort analysis. Int. J. Environ. Res. Public Health.

[34] Jee SH, Golub JE, Jo J, Park IS, Ohrr H, Samet JM (2009). Smoking and risk of tuberculosis incidence, mortality, and recurrence in South Korean men and women. Am. J. Epidemiol..

[35] Wen CP, Chan TC, Chan HT (2010). The reduction of tuberculosis risks by smoking cessation. BMC Infect. Dis..

[36] Imtiaz S, Shield KD, Roerecke M, Samokhvalov AV, Lönnroth K, Rehm J (2017). Alcohol consumption as a risk factor for tuberculosis: Meta-analyses and burden of disease. Eur. Respir. J..

[37] Ansot Anzats Ruphine P, Oscar NK, Bongo GN, Lukelo Désiré N, JeanPierre AJ (2019). Risk factors for mortality in patients with TB/HIV co-infection at the General Provincial Reference Hospital of Kinshasa, the Democratic Republic of the Congo. Arch. Intern. Med. Res..

[38] Zerbini E, Greco A, Estrada S, Cisneros M, Colombo C, Beltrame S, Boncompain C, Genero S (2017). Risk factors associated with tuberculosis mortality in adults in six provinces of Argentina. Medicina.

[39] Tuberculosis National Programme, C., Group, C. T., & Secretariat, P. National Tuberculosis. 9. (2014). http://www.nationalplanningcycles.org/sites/default/files/planning_cycle_repository/cameroon/nationaltuberculosisstrategicplan2010.pdf. (Accessed Feb 2021).

[40] United Nations Office for Project Services. DRC: national TB program implements intensified TB case-finding approach in Kinshasa. The Strategic Initiative to Find the Missing People with TB. (2018). https://stoptb-strategicinitiative.org/index.php/2019/04/15/drc-national-tb-program-implements-intensified-tb-case-finding-approach-in-kinshasa/. (Accessed Feb 2021).

[41] Programme national de lutte contre la tuberculose.pdf. http://www.apf.francophonie.org/IMG/pdf/9._programme_national_de_lutte_contre_la_tuberculose.pdf. (Accessed Feb 2021).

[42] OCHA services Reliefweb. Improving TB treatment and control in West and Central Africa. (2018). https://reliefweb.int/report/world/improving-tb-treatment-and-control-west-and-central-africa. (Accessed Feb 2021).

[43] Trading economics. Chad GDP Annual Growth Rate. (2019). https://tradingeconomics.com/chad/gdp-growth-annual. (Accessed Feb 2021).

[44] Trading economics. Cameroon GDP Annual Growth Rate. (2019). https://tradingeconomics.com/cameroon/gdp-growth-annual. (Accessed Mar 2021).

[45] Trading economics. Central African Republic GDP Annual Growth Rate. (2019). https://tradingeconomics.com/central-african-republic/gdp-growth-annual. (Accessed Mar 2021).

[46] Statista. The Democratic Republic of the Congo: Gross domestic product (GDP) per capita in current prices from 1985 to 2025. (2020). https://www.statista.com/statistics/1041557/gross-domestic-product-gdp-per-capita-in-the-democratic-republic-of-the-congo/. (Accessed Mar 2021).

[47] Miele K, Bamrah Morris S, Tepper NK (2020). Tuberculosis in Pregnancy. Obstet. Gynecol..

[48] Survey H (2009). Democratic republic of Congo 2007: Results from the demographic and health survey. Stud. Fam. Plann..

[49] Winston CA, Navin TR (2010). Birth cohort effect on latent tuberculosis infection prevalence, United States. BMC Infect. Dis..

[50] Musa BM, Adamu AL, Galadanci NA, Zubayr B, Odoh CN, Aliyu MH (2017). Trends in prevalence of multi drug resistant tuberculosis in sub-Saharan Africa: A systematic review and meta-analysis. PLoS One.

[51] Nicola M, Alsafi Z, Sohrabi C, Kerwan A, Al-Jabir A, Iosifidis C, Agha M, Agha R (2020). The socio-economic implications of the coronavirus pandemic (COVID-19): A review. Int. J. Surg. (Lond. Engl.).

[52] Gao D, Zou Z, Zhang W (2020). Age-period-cohort analysis of HIV/AIDS mortality in China: Data from the Global Burden of Disease Study 2016. Sci Rep.

[53] Martial NT, Mubarik S, Yu C (2021). The trend of HIV/AIDS incidence and risks associated with age, period, and birth cohort in Four Central African Countries. Int. J. Environ. Res. Public Health.

